# Coastal cultural ecosystem services and adolescents’ subjective well-being

**DOI:** 10.1007/s13280-024-02043-2

**Published:** 2024-06-13

**Authors:** David Cabana, Stefania Pinna, Simone Farina, Daniele Grech, Nicholas Barbieri, Ivan Guala

**Affiliations:** 1 IMC - International Marine Centre, Loc. Sa Mardini Torregrande, 09170 Oristano, Italy; 2 Climate Service Center Germany (GERICS), Helmholtz-Zentrum Hereon, Fischertwiete 1, 20095 Hamburg, Germany; 3 GREEN LEAF - Groupe de Recherche en Education à l’Environnement et à la Nature, Laboratory of Affective Ecology, Università della Valle d’Aosta, Strada Cappuccini 2a, 11100 Aosta, Italy; 4 Department of Integrative Marine Ecology, Genoa Marine Centre, Stazione Zoologica Anton Dohrn - National Institute of Marine Biology, Ecology and Biotechnology, Villa del Principe, Piazza del Principe 4, 16126 Genoa, Italy; 5 National Research Council, Institute for the Study of Anthropic Impacts and Sustainability in the Marine Environment (CNR-IAS), Loc. Sa Mardini, 09170 Torre Grande, OR Italy

**Keywords:** Coastal management, Cultural and recreational activities, Human well-being, Interdisciplinary, Nature connectedness, Teenagers

## Abstract

Despite growing recognition of nature’s impact on mental health, its specific effects on adolescents remain unclear. This gap hinders effective strategies for youth well-being in a world facing increasing environmental pressures. This study directly investigates the connections between coastal environments and subjective well-being in adolescents. We explore how interactions with coastal landscapes and associated cultural ecosystem services contribute to both personal and social dimensions of well-being in a sample of 202 adolescents (16 and 17 years old) from Sardinia, Italy. Our findings reveal a beneficial impact of coastal experiences, promoting positive affect, social integration, and physiological well-being. However, human pressures on these environments can lead to negative affect, while fostering a sense of social contribution. This research highlights the complex interplay between coastal environments, human impacts, and teenagers’ well-being. Understanding these links empowers planners to craft coastal management strategies that balance adolescents’ well-being with the long-term sustainability of coastal regions.

**Supplementary Information:**

The online version contains supplementary material available at 10.1007/s13280-024-02043-2.

## Introduction

To ensure sustainable development in the face of escalating environmental threats, the conservation and restoration of ecosystem services are paramount (MEA 2005). Recognising this, international agendas like the United Nations (UN) 2030 and the European Strategy for 2030 highlight the critical role of nature in promoting physical, mental, and social well-being, particularly in the face of global challenges (United-Nations 2015; European-Commission 2020). Similarly, the World Health Organization emphasises the importance of preserving ecosystems for safeguarding health and well-being (WHO 2021). This growing recognition has fuelled research exploring the potential for natural environments to positively influence various aspects of health and well-being. Building on this foundation, prior studies established connections between experiences in nature and enhanced psychological well-being, including increased positive mood and reduced negative emotions like anxiety and anger, making happier and healthier people (Russell et al. 2013; Bratman et al. 2015; Capaldi et al. 2015). These results emphasise the multidimensional benefits of nature exposure across the lifespan, promoting both mental and physical health for all ages. While research on adults has shown benefits, understanding the full impact on adolescents, a critical developmental stage characterised by significant physical, cognitive, and emotional changes requires dedicated research (Suzanne et al. 2018; Jackson et al. 2021). This unique window of opportunity, as Dahl (2004) suggests, presents valuable insights into the influence of nature exposure. Existing research on teenagers and outdoor environments also points towards psychological benefits like improved mood, positive development, and competence, alongside physiological benefits like lower blood pressure, increased physical activity, reduced risk of cardiovascular disease, and better sleep (Jimenez et al. 2021). Despite this wealth of evidence, a critical gap remains in understanding how teenagers, undergoing significant physical, cognitive, and emotional changes, interact with and benefit from the natural world (Bray et al. 2022).

While a substantial body of literature explores the benefits people receive from interacting with nature, the focus often centres on specific environments like forests and urban green spaces (Reece et al. 2021). This leaves a gap in our understanding of the potential benefits offered by “blue spaces”. Coastal environments are dynamic social–ecological systems (SESs) where diverse ecosystems (e.g., estuaries, salt marshes, sandy beaches, and rocky shores) and human societies are intricately linked (Rendón et al. 2019). This is especially true for islands, where communities depend heavily on the health of surrounding marine environments (Forster et al. 2014). Understanding these interdependencies is vital for the long-term sustainability of these unique places (Uehara et al. 2019). Coastal environments with high biodiversity and scenic beauty provide distinct interactions with nature, such as exposure to the vastness of the ocean, the sound of waves, and the dynamic character of the shoreline. These unique features warrant further investigation, especially regarding adolescents’ mental well-being. Recent studies even suggest that childhood exposure to blue spaces may have long-term benefits in adulthood, highlighting the need for more scientific exploration in this area (Vitale et al. 2022).

As we delve deeper into these people nature connections, a multidisciplinary approach is necessary to achieve a more comprehensive understanding of human–environment interactions. This approach has been successfully employed in the concept of ecosystem services, which utilises frameworks and theories from multiple disciplines to analyse how nature benefits humans (Pascual et al. 2017; Haines-Young and Potschin 2018). The concept of cultural ecosystem services (CESs) exemplifies this approach. CES encompasses the spiritual, educational, and recreational values that nature provides alongside more tangible benefits like food and water (Chan et al. 2012; Daniel et al. 2012; Hernández-Morcillo et al. 2013). Research on coastal CES utilises multidisciplinary methods for assessment, quantification, and mapping (Ahtiainen et al. 2019; Cabana et al. 2020; Ruiz-Frau et al. 2020). These efforts not only provide insights into the human connection with coastal environments but also inform coastal management strategies (Gee et al. 2017; Blake et al. 2021; Dou et al. 2021).

Despite a surge in CES research, a critical gap exists from a management standpoint. Current studies often fail to connect CES to real-world impacts on human well-being, particularly subjective well-being (SWB)—how people experience their life (Bratman et al. 2019; Kosanic and Petzold 2020; Nowak-Olejnik et al. 2022). This disconnect is especially concerning for adolescents, who stand to benefit greatly from the unique opportunities coastal environments offer (Bray et al. 2022). To bridge this gap and inform effective management strategies, we need to integrate human experiences and the impact of our activities directly into CES research. Effective coastal planning requires incorporating diverse perspectives (Simpson et al. 2016). Stakeholders hold a range of economic, cultural, and social values concerning coastal ecosystems. To navigate these differences and manage potential conflicts, robust public participation is crucial (Garcia Rodrigues et al. 2017; Veidemane et al. 2024).

This research addresses the gap by investigating how adolescents in Sardinia interact with coastal environments, how these interactions depend on CES, and ultimately contribute to their SWB. Sardinia exemplifies this link due to its unique dependence on healthy coastal ecosystems for tourism and the well-being of its inhabitants. By focusing on youth (16–17 years old) engaging in leisure and cultural activities by the coast, we examine the interplay between these activities, CES, and personal and social SWB. This approach addresses the underrepresentation of adolescents’ voices in CES and coastal well-being research, a critical gap identified by Reece et al. (2021), which is crucial for developing targeted interventions and policies (Wright and McLeod 2014).

## Materials and methods

### Study area

Sardinia, a large Mediterranean island (24,100 km^2^), boasts a diverse coastline. Rocky shores, sandy beaches, coastal lagoons, and artificial areas characterise its coastal ecosystems and landscape. The island’s rich marine environment is further protected by numerous terrestrial and marine conservation areas. Unlike other Mediterranean regions, Sardinia’s coastline remains relatively undeveloped (Pungetti et al. 2008). The population, circa 1.6 million, is scattered across rural and semi-urban settlements. This rural character is evident in the prevalence of small towns and villages distributed in clusters (average population density 60 inhabitants/km^2^). Coastal communities range from traditional fishing villages to more industrialised centres, with tourism acting as a major economic driver, particularly during summer months (Ioppolo et al. 2013). Sardinia’s unique characteristics make it a compelling case study for investigating nature connectedness in adolescents. The island’s diverse coastal environments offer a range of natural spaces for teenagers to interact with. Thus, studying adolescents in Sardinia can provide valuable insights into the connection between nature exposure and well-being during this critical developmental stage.

### The survey questionnaire

Informed by local social scientists, ecologists, and a schoolteacher, we designed a semi-structured survey questionnaire to capture the expression of values relevant to participants' experiencing the coast. This approach combines closed-ended questions with ticking boxes and open-ended sections that allows participants to provide additional details and enable us to draw connections between the different studied elements—Elements of the environment, cultural and recreational activities, CES, and SWB (refer to “Supplementary Material”). For the CES classification, we ensured alignment with the widely used Common International Classification of Ecosystem Services (CICES) for consistency across Europe (Haines-Young and Potschin 2018).

To investigate the link between adolescents’ coastal interactions, CES, and SWB in Sardinia, we surveyed from January 2019 to January 2020. We intentionally focused on a specific age group (16–17 years old) by distributing individual paper-based questionnaires to student groups visiting the International Marine Centre in Sardinia, Italy, or scientists visiting the schools. These groups came from eight public schools across the region, ensuring a diverse geographical representation.

### Accounting well-being

We used a multifaceted approach to assess adolescents’ well-being in coastal environments. This approach combined the strengths of two established frameworks:

Dieners et al. Subjective Well-being: This framework employs self-reported measures to capture immediate well-being impacts through positive/negative affect and life satisfaction. It provides a standardised method for understanding subjective experiences in coastal settings (Diener et al. 2006, 2018).

Keyes' Model of Social Well-being: This model goes beyond individual feelings, examining how coastal environments influence adolescents sense of social integration, contribution, and connection to their communities Keyes (1998).

By combining these frameworks, the proposed approach offers a comprehensive and culturally adaptable perspective. Diener’s framework provides a universal foundation, while Keyes' model allows us to explore potential cultural influences on participants' social experiences (Diener et al. 2018; Sim and Diener 2018; Jebb et al. 2020). Together, these well-established tools provide foundation for investigating the relationships between coastal environments, cultural activities, and both personal and social well-being in adolescents.

### Human pressures

The proposed conceptual model also takes into account human pressures on the environment, allowing us to explore potential negative synergies beyond the positive aspects of human–nature interactions (Huynh et al. 2022). To assess these pressures, we employed open-ended questions where students could share their concerns regarding the impact of human activities on cultural activities, CES, and well-being.

The qualitative approach prioritised capturing participants’ life experiences and personal values related to the coast. To facilitate this, we employed semi-structured interviews with open-ended questions. This approach is balanced providing some structure with ticking boxes (details in “Supplementary Material”) to guide the conversation, while also allowing ample space for participants to elaborate on their responses.

### Data collection

Data collection involved two phases: a group familiarisation session and a subsequent response to individual questionnaires. The first part was a 30-min group introduction session. This session began with a 20-min introduction to the ecosystem services framework and its overall purpose. To provide context, we then presented a definition of the coast, accompanied by visuals showcasing the diverse coastal ecosystems found throughout Sardinia. Following this introduction, we facilitated a 10-min open discussion where students (in groups of approximately 25) were invited to share their experiences or encounters with these coastal environments. This interactive discussion aimed to familiarise students with the topics and encourage them to share their perspectives, minimising the potential for bias in their later responses. Importantly, no data were collected during this familiarisation phase. Following the familiarisation session, participants completed an individual questionnaire. This questionnaire included semi-structured and open-ended questions designed to capture information on cultural activities, CES, and SWB (see “Supplementary Material”). The anonymous survey, conducted in collaboration with schools, adhered to a pre-established protocol requiring parental permission for research-related questionnaires. The survey strictly abstained from collecting any personal information, ensuring compliance with the EU General Data Protection Regulation (GDPR) (European-Parliament 2016). Yet, parental consent was considered an ethical necessity.

### Data analysis

To understand adolescents' interactions with the coast and their impact on subjective well-being (SWB), we employed an inductive thematic analysis approach. This qualitative method allows themes and concepts to emerge directly from participants’ responses, emphasising their lived experiences (Braun and Clarke 2006). The analysis focused on identifying associations between participants' descriptions of cultural and recreational activities, the CES they derived from these activities, and their SWB (Diener et al. 2006; Bell et al. 2015).

For open-ended questions, we used a line-by-line coding approach followed by a “meaning condensation” (Marshall and Rossman 2014). This involved identifying units of meaning (words and concepts) and assigning them to relevant categories and subcategories based on the open-ended questions. As patterns emerged, through this iterative process, similar categories were grouped into broader themes or concepts. This ensured that the final themes accurately reflected the lived experiences recounted by the participants accommodating the different studied elements.

By systematically coding and grouping responses based on these themes, we were able to gain a deeper understanding of several key aspects:

- How teenagers engage with the coast through various cultural and recreational activities
- What CES they value most within coastal environments
- How these interactions with the coast influence their SWB across different dimensions
- Human pressures

This thematic analysis ultimately allowed us to develop a conceptual model (presented in the Results section) that explores the links between coastal environments, cultural activities, and various aspects of adolescent well-being.

## Results

### Socio-cultural variables

**Fig. 1:**
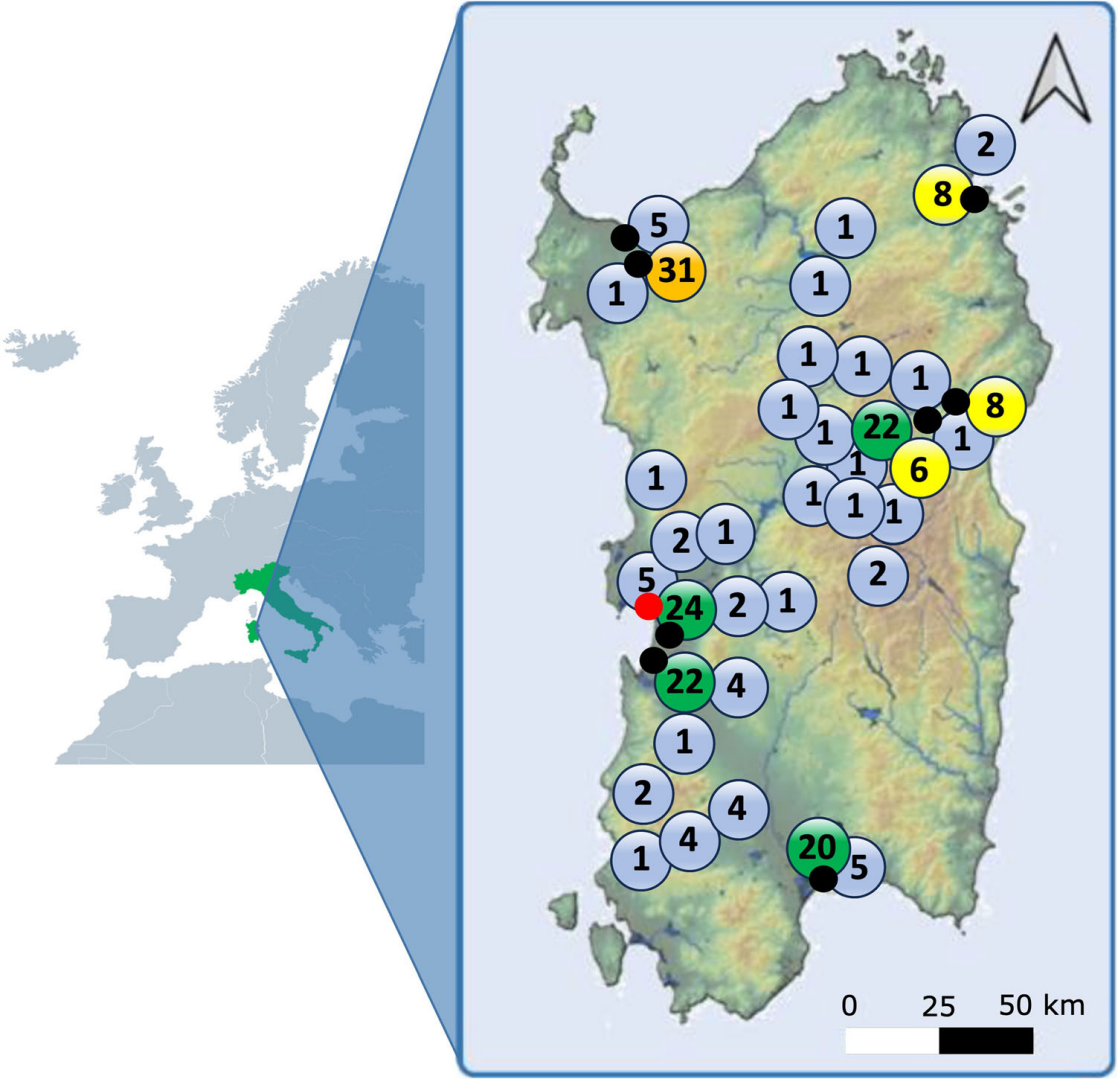
The map displays the distribution of survey participant’s residences (numbered circles), the locations of participating schools (black dots), and the International Marine Center (red dot) in Sardinia, Italy. The circle colour shows the number of respondents per municipality. Blue: 5 or fewer respondents. Yellow: 6–10 respondents. Green: 11–30 respondents. Orange: more than 30 respondents

We analysed 202 questionnaires responded by local students between 16 and 17 years (128 females and 74 males) with residences across the island of Sardinia (Fig. 1).

### Links between environmental factors, CES, and cultural and recreational activities

**Fig. 2:**
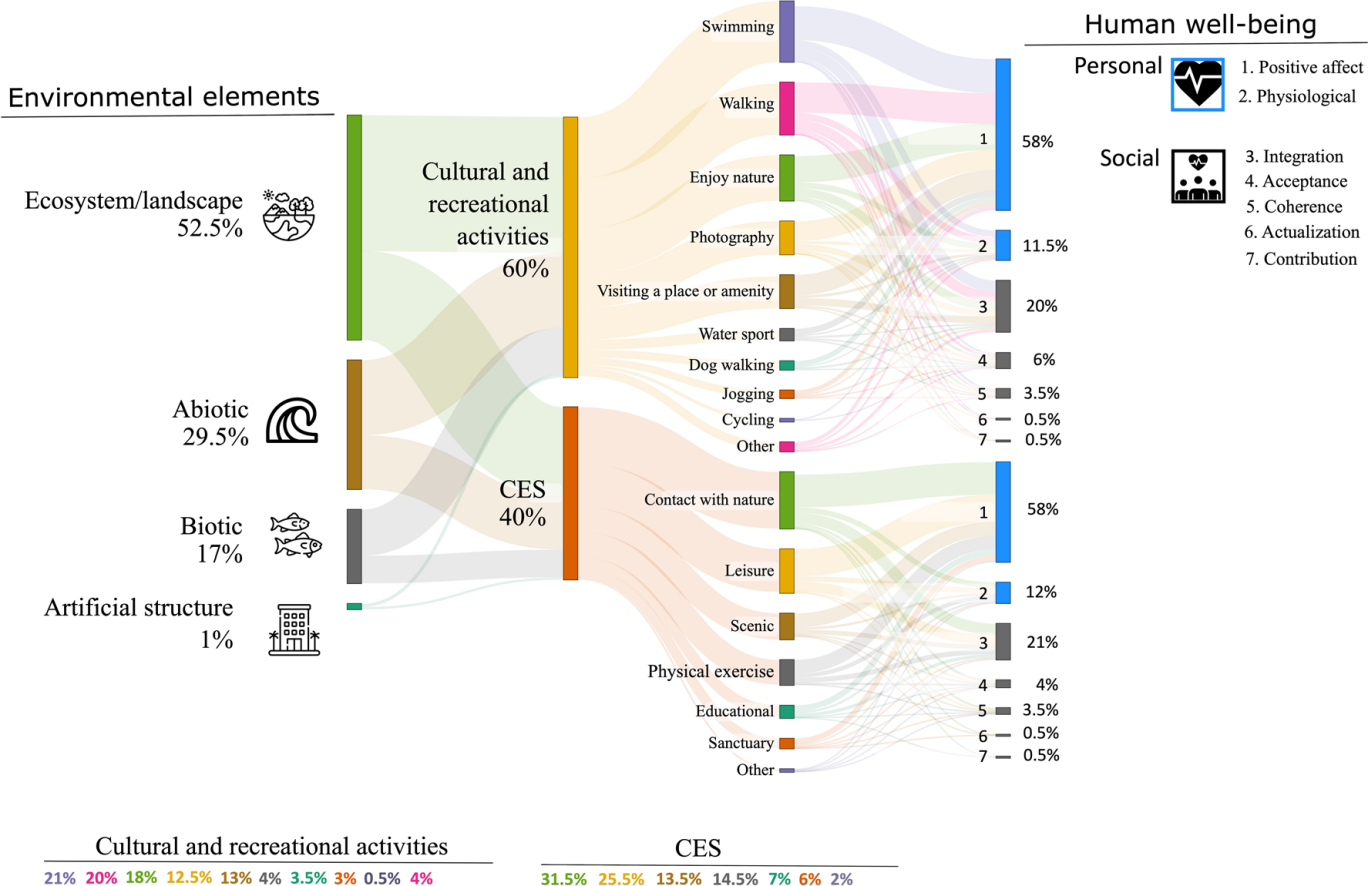
The Sankey diagram presents the synergies among the different analysed components. The flows should be read from left, “environmental elements”, to right, “components of SWB”. The diagram demonstrates how environmental factors (ecosystems, abiotic, biotic, and artificial structures) underpin cultural and recreational activities and CES, which contribute to both types of SWB (i.e. social and personal). Later, these links and flows are instrumental to draft the conceptual model

Analysis of open-ended survey questions (“Supplementary Material”) revealed diverse aspects of participants’ engagement with the coast (Fig. 2). Responses primarily adopted an ecosystem and landscape viewpoint, with references as “breath-taking landscapes”. Participants then focused on abiotic elements, mentioning attributes like “crystal clear waters” and “the sound of the waves”. Biotic factors followed, including mentions of “plenty of fish to watch” and “lively *Posidonia* meadows”. Artificial structures were rarely mentioned, with only a few references as “beautiful promenades”.

A deeper examination of the responses showed while reflecting on their coastal engagement participants also considered different CES (40%) and cultural and recreational activities (60%) (Fig. 2). For example, one participant stated, “I love visiting the coast because it relaxes me, and I get to enjoy beautiful days swimming with my friends”. This quote exemplifies the connection between the coastal environment, relaxation as a CES, and the recreational activity of swimming.

### Quantification of the links between CES and SWB

Analysis of the semi-closed question on CES revealed that respondents attributed the highest importance to “contact with nature", as shown in Fig. 2. Notably, while this question did not explicitly prompt acknowledgement of other CES benefits like scientific knowledge or a sense of place, these themes emerged in later open-ended questions. For example, participants expressed sentiments with strong connotations of sense of place, such as: “I value the natural condition, characterised by the origins where the culture of our region is also rooted” or “I appreciate the pride of the people living here and our passion for the territory and nature” or “In Sardinia, we have one of the most beautiful seas”.

Using coded terms and inductive allocation techniques, further insights were obtained to quantify the connections between the identified CES and SWB, specifically focusing on various facets of personal and social well-being components (Table 1). The results highlighted the strongest connection between CES and positive affect, social integration, and physiological well-being (Fig. 2). Although not a primary focus, the open-ended questions offered the potential to gain insights into aspects of physiological well-being. In this study, physiological well-being refers to the self-evaluation of an individual’s physical health and functionality (Haluza et al. 2014). For example, one respondent wrote “I like to be in contact with nature under the sun to absorb Vitamin D, the sea also helps blood circulation…”.

**Table 1:** Personal and social well-being components identified from open-ended survey questions

SWB	Type	Concepts and terms	Examples
Personal	Positive affect	Relaxation, calmness, joy, disconnection, peace, free mind, serenity, and love	“For me, the transparent waters transmit peace, freedom, and tranquilly”, or “Nature influences people a lot, especially temperament and mind. I think observing a beautiful beach with clean water, and white sand will improve their mood, contrary to what would happen on a dirty beach”, or “**I am happy to see how new restrictions are being imposed to prevent people trampling on the dunes**.”
	Negative affect	Negative emotions associated with frustration or worry are mainly related to environmental problems and people’s behaviour. Include elements associated with frustration, discontent, worry, and irritation	"**I do not like tourism; it is out of control, like colonialism. Carelessness causes environmental disasters…**”**,** or “**Yes, the fact that there are more tourists on our beaches makes it increasingly difficult to find the tranquillity and peace that I would like to find.**” and “**I am disappointed because I perceive little respect of people towards the beaches. For example, in Spiaggia Rosa there are people, even Sardinians, picking up sand as a keepsake or who knows what…**” or “**I do not like to swim when I find garbage on the water, it could be dangerous**”
	Physiological well-being	The individual physical well-being involves multiple components related to the body’s condition. These components encompass overall physical fitness, the absence of illnesses, and the proper functioning of bodily systems. (e.g., boost of Vitamin D or blood circulation)	“It is healthy for the body. Some time ago I had breathing problems and visiting the coast and swimming helped me recover”, “I like to be in contact with nature under the sun to absorb vitamin D, the sea also helps blood circulation…” and “I like to relax and sunbathe. What’s more, the sea is good for my health, and the landscape is beautiful”
Social	Social integration	The quality of one’s relationship to society and community (e.g., family, friends, acquittances, and my people). The coast seen as a space for social integration	"Being at the coast is about me, nature and friends”, “I like the contact with nature because it frees the mind from being overwhelmed with thoughts. I like to be with my people breathing pure air”
	Social coherence	Includes aspects such as participation in social life. Being part of nature and society. The coast seen as a space contributing to social coherence	“I like that the coast represents a fundamental meeting point for culture and the people of the island”, or “Mankind sometimes needs to feel part of nature”. “**I try to make people think about these problems (marine litter), and generally, people appreciate knowing about it**”
	Social acceptance	The coast seen as a space contributing to social acceptance. Trusting others and holding positive opinions about groups and people	“The coast connects me to the Sardinian people” or “Sardinia has a diverse nature, I feel extraordinarily strong cultural values and tradition”
	Social contribution	Evaluation of one’s social value. Seeing one’s own daily activities as useful to and valued by society—contributing to the natural environment	“**When visiting the beach, I am careful not to take any sand. Also, I ask my friends and other people not to leave rubbish around**”**,** “**Now I do not step over the dunes…**” or “**…when possible, I help clean the beach**”
	Social actualisation	Involves the potential and direction of societal development. It acknowledges the capacity for individuals, groups, and society to progress positively. It encompasses self-fulfilment, personal growth within social interactions, contributing to communities, and realising individual potential within a broader societal context	“I have understood that we should just behave as observers and treat the natural environment as something sacred, keeping it for future generations”

This table presents examples of personal and social well-being categories and subcategories identified from the analysis of open-ended survey questions. The components reflect participants' perceptions of their engagement with the coast, associated values, and the most compelling human pressures they identified. Responses directly related to human pressures are shown in **bold** text

Interestingly, no elements related to negative affect were documented in the participants' responses regarding the associations between CES and SWB.

### Links between cultural and recreational activities and SWB

Analysis of the semi-closed questions on cultural and recreational activities revealed the high value participants placed on the coast (Fig. 2). Qualitative analysis of the open-ended responses further emphasised the appreciation for the coast as a safe and calming environment, for social interaction. For example, one respondent stated, “The waters are very calm, and I can relax, run, swim, spend the time, and enjoy myself with my family, friends, and acquittances”.

To quantify the connections between the coastal cultural and recreational activities and SWB, we employed coded terms and inductive allocation to categorise response within personal and social well-being components (Table 1). The results highlighted positive affect, social integration, physiological well-being, and social acceptance as the primary contributors from cultural and recreational activities (Fig. 2). Notably, no components of negative affect appeared when participants reflected on the association between these activities and SWB.

### Links between human pressures and SWB

**Fig. 3:**
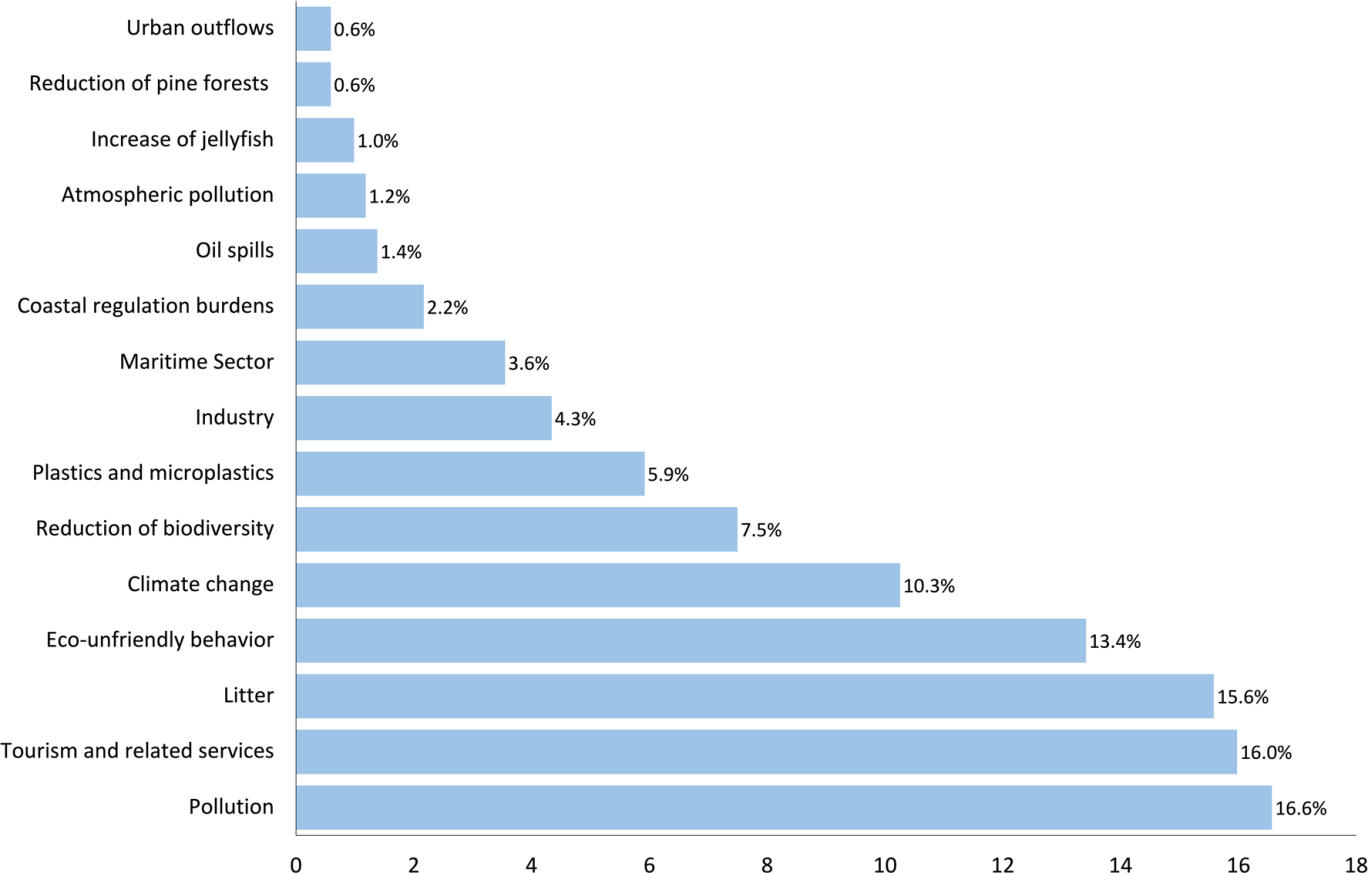
Responses to the question on human pressures affecting the state of health of the coast in Sardinia

Perceived human pressures with general pollution as the most relevant were explored for their connection to SWB through analysis of open-ended responses. This analysis revealed synergies between human pressures and negative affect (53%) (like frustration with pollution or overcrowding); they can also inspire people to act and contribute to positive change and social contribution (42%), (like volunteering for beach clean-ups or advocating for sustainable practices). Other aspects as positive affect, social actualisation, and social coherence had minimal individual contributions (around 1% each) (Table 1, Fig. 3).

### Integrated analysis

**Fig. 4:**
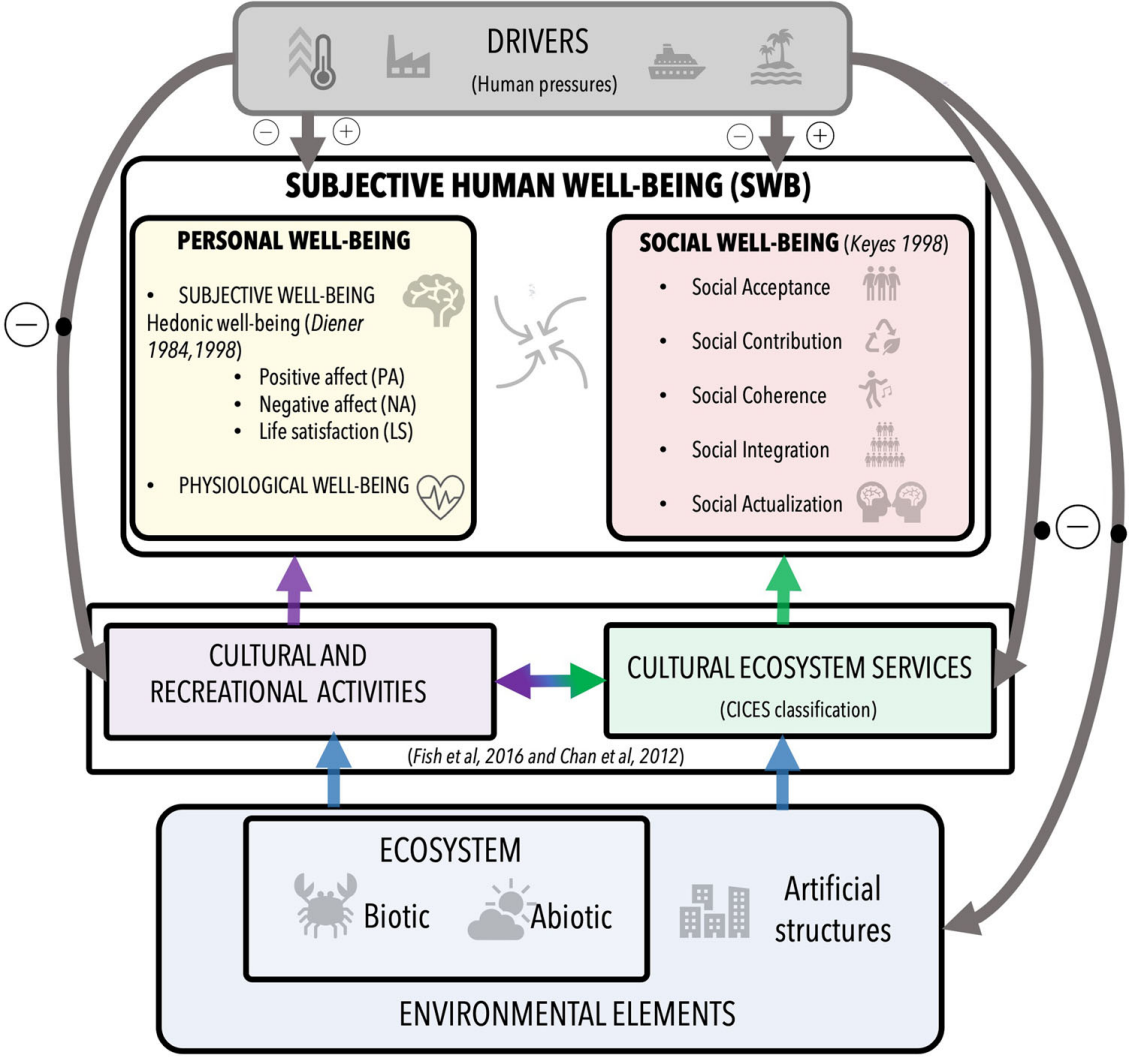
A Model of Coastal Environments and Adolescents Subjective Well-being: This conceptual model explores how coastal environments, cultural activities, and cultural ecosystem services (CESs) influence adolescents' subjective well-being (SWB). It highlights the interconnectedness of these elements and acknowledges the potential impact of human actions on all aspects of the system. Human Pressures: Human activities can have negative (−) impacts on environmental elements, cultural and recreational activities, and cultural ecosystem services (CESs). Based on the responses to the survey, the model acknowledges the dual influence of cultural activities on SWB. Positive (+) effect includes social contribution. Negative (−) effect can include stress or anxiety associated with certain human pressures

The overall analysis offers an integrated perspective on the interconnectedness between the coastal environment, human pressures, CES, and cultural/recreational activities, all contributing to SWB (Fig. 4). The central argument is that environmental conditions and cultural/recreational activities linked to CES collectively contribute to personal and social SWB. CES functions as a pathway for individuals to derive means by which individuals derive personal and social SWB while acknowledging the reciprocal effects of human pressures on the environment.

The analysis highlights the connections between environmental factors that provide CES and the opportunities for cultural and recreational activities. These factors often overlap significantly, with a high degree of interaction (Fish et al. 2016). While the concept of CES might not be readily apparent to participants, their responses often describe coastal engagement through activities and feelings. In this sense, the analysis bridges the gap between CES and cultural/recreational activities, ultimately connecting them to various SWB elements.

Furthermore, the analysis identifies the links between human pressures, the natural environment, CES, cultural activities, and SWB. Our findings contribute to articulating SWB elements and often considered separate from CES assessments and framework (Kosanic and Petzold 2020; Huynh et al. 2022; Nowak-Olejnik et al. 2022).

## Discussion

This section dissects the intricate link between adolescents (16 and 17 years old) and Sardinia’s coastal environment, pinpointing how it influences their subjective well-being (SWB). Through a cultural ecosystem service (CES) lens, we explore how environmental elements, human pressures, and adolescent interactions with these environments shape both personal and social aspects of SWB. This approach addresses the underrepresentation of adolescents' voices in CES and coastal well-being research (Reece et al. 2021), a critical aspect for developing targeted coastal management interventions and policies (Wright and McLeod 2014).

### Unveiling adolescents’ preferences for natural coastal environments

This study explored the environmental factors that adolescents recognise as important for coastal CES and cultural/recreational activities (Fig. 4). In this study as in Ruiz-Frau et al. (2020), our findings revealed a preference for natural elements over man-made structures or urban environments. This highlights a general desire for interaction with the coast in its natural state. People, regardless of age, appear to appreciate the inherent beauty of natural coastlines over developed ones. This suggests a general preference for experiencing the coast in a more natural setting (Yuan et al. 2023).

Interestingly, adolescents’ responses emphasised the value that they associate with specific landscape features (e.g., coast, beach, dunes, and pine forest) and abiotic factors (e.g., “breath-taking landscape”, “crystal clear waters…”, “white sand” or “there is nothing more relaxing than the sound of the waves). While other studies suggest that values can vary geographically, across coastal ecosystems and with environmental literacy (Ruiz-Frau et al. 2020; Zunino et al. 2020), our findings align with the broader consensus that contact with nature, leisure, and scenic beauty are key coastal CES (Ahtiainen et al. 2019; Retka et al. 2019; Blythe et al. 2020). This research found a unique pattern—adolescents placed a higher value on physical exercise than scenic beauty when enjoying the coast. Conversely, educational and spiritual values received less emphasis, potentially linked to the types of activities adolescents engage in at the coast, which can vary depending on the specific coastal area and ecosystem (Cabana et al. 2020). This highlights the importance of considering adolescents’ perspectives when understanding their preferences for coastal experiences.

### Unveiling adolescents' perspectives on coastal well-being

Exposure to coastal environments has documented benefits for mental health and well-being in adults (Gascon et al. 2017) and children (Vitale et al. 2022). Our results emphasise a complex relationship between CES, cultural activities, and SWB (Fig. 4, Table 1). These elements interact, influencing well-being depending on the nature and strength of their connections.

Adolescents' experiences with the coast highlight positive affect (relaxation, calmness, and happiness) as a key SWB aspect. This is evident in responses like “The sound of the waves, the sand between my toes, staring at the horizon heals me.” These findings align with research prioritising positive affect over physical benefits when considering nature interactions in peri-urban areas (Wangai et al. 2017). However, the interconnectedness between emotional and physical well-being is acknowledged. Studies suggest links between emotional and physical health indicators following nature-based interventions in coastal areas (Maund et al. 2019). Physical activities by the coast can create a space for self-connection and connection with nature, potentially impacting adolescents’ ability to cope with challenges and influencing their future health (Kessler et al. 2007; Nowak-Olejnik et al. 2022; Vitale et al. 2022).

While social well-being received less emphasis, adolescents’ narratives indicate the importance of social integration, acceptance, and coherence. Social integration, often linked to family and friends, is evident in responses like “Being at the coast is about me, my friends, and nature.” Social acceptance, related to a sense of belonging, is recognised as a space for cultural integration like “The coast connects me to Sardinian people”. This is particularly relevant for island communities where territorial separation fosters strong cultural identity (Coulthard et al. 2017; Freeman et al. 2022). Finally, social coherence emerges as the coast being perceived as a space for a participatory social life (Fig. 4, Table 1).

### Adolescents' concerns: Human pressures and well-being

While coastal interactions offer positive connections between cultural activities, CES, and SWB, they are also threatened by human pressures like climate change, pollution, and algal blooms (Fleming et al. 2014). This study of adolescents found that these pressures primarily influence personal SWB through increased negative affect but also lead to a greater emphasis on social contribution.

Human activities as tourism and environmental issues like pollution and littering were identified as significant concerns. Adolescents highlighted the emotional distress caused by these issues, which disrupt the natural beauty of the coast and limit recreational opportunities. Responses like “tourism is overwhelming and unregulated” reflect a perception that excessive tourism disrupts the tranquillity adolescents seek at the coast. This disrupts experiences crucial for their well-being, such as relaxation, stress reduction, and finding inspiration in nature (Willis et al. 2018). Interestingly, research suggests tourist view CES as a positive influence on their own well-being (Willis 2015). This highlights the complex and sometimes conflicting perspectives surrounding coastal experiences.

Coastal pollution and littering were additional concerns, fostering feelings of disappointment and a sense that the environment is not respected, like “I feel people have little respect for the beaches”. However, adolescents also expressed social responsibility through pro-environmental behaviours like “I tell my friends and others not to litter.” This link between human pressures and social contribution underscores the importance of environmental stewardship. Considering semi-urban environment where adolescents often live in areas where nature is central to their lives they likely have a strong connection to nature (Berto et al. 2015; Mackay and Schmitt 2019; Pritchard et al. 2020). This complex relationship between human pressures, pro-environmental behaviour, and SWB highlights the need for future interdisciplinary research to explore the human dimensions of coastal areas. Additionally, future research should focus on the mechanisms linking the natural environment, CES, and adolescents' well-being at the coast.

### Limitations and future research

The current study design, encompassing a broad geographic region, restricts the generalisability of the findings to specific locales. Future investigations could mitigate this limitation by adopting a municipality-level focus. Employing density and hotspot mapping techniques would facilitate the identification of areas harbouring unique value for adolescent populations. This granular approach would directly inform the development of targeted and impactful coastal management strategies at the local level. Furthermore, while the study successfully illuminates adolescent experiences within coastal environments, its overall robustness could be enhanced through validation across diverse age demographics and geographic regions. A broader validation process would not only bolster the generalisability of the findings but also strengthen the argument for incorporating the perspectives of all age groups into coastal management plans. Finally, the transition to digital data collection and analysis, particularly for open-ended questions, presents an opportunity to optimise efficiency and potentially unlock deeper insights into adolescent perceptions. Digital solutions could streamline the process of identifying and condensing meaning units from open-ended responses, thereby enabling a more nuanced understanding of the entire spectrum of adolescent experiences within coastal settings.

## Conclusions

This study explored the connections between coastal environments, cultural activities, human pressures, and adolescent subjective well-being (SWB) through a cultural ecosystem service (CES) lens. The findings provide insights for enhancing coastal planning and management, while also highlighting areas for future research.

Sustainable coastal management requires recognising the unique needs and preferences of all stakeholders, including adolescents. Their experiences with the coast, particularly on islands, shape their connection to the environment (Kjørholt and Bunting 2023; Kjørholt et al. 2023). Coastal planning should prioritise designated spaces for adolescent activities and the preservation of natural features that not only offer recreation but also foster a deeper environmental connection.

Adolescents' narratives about their favourite coastal spots, memories, and aspirations can guide planners in creating inclusive and vibrant coastal spaces. Furthermore, integrating adolescents into the planning process fosters a sense of ownership. Social engagement through local activities, environmental volunteering, and advocating for their needs allows them to exercise rights and responsibilities within the community. Strong local social identity—feeling connected to the place—enhances happiness, well-being, and place attachment (Maricchiolo et al. 2021). When adolescents feel part of the decision-making process, they are more likely to become responsible stewards of the coast. This inclusive approach ensures that coastal planning reflects the voices of all age groups, fostering a sense of pride and collective responsibility for the future.

Capturing teenagers' voices revealed previously unidentified aspects of the coastal experience for this age group. For instance, adolescents expressed a negative affect associated with excessive tourism, highlighting a concern not previously documented in this age group. This underscores the importance of considering teenagers' perspectives on cultural significance and recreational value when developing coastal management strategies. Furthermore, integrating both personal and social aspects of SWB provided a comprehensive understanding of how coastal experiences contribute to well-being. This knowledge allows for the creation of management plans that promote positive experiences, such as relaxation and stress reduction in nature, and mitigate negative impacts like overcrowding.

The research also introduces a conceptual model exploring the linkages between coastal environments, CES, and adolescent SWB. Understanding these connections allows coastal managers to develop more effective and inclusive strategies that prioritise both environmental health and the well-being of coastal communities across all age groups. Recognising the link between adolescent well-being and the coastal environment allows for the development of sustainable management practices that promote a positive relationship between teenagers and the environment. Integrating metrics for coastal management tools’ contribution to adolescent well-being and ecosystem services offers a more nuanced understanding of their effectiveness. This data can be used to refine and improve future coastal management plans, fostering sustainable practices that support both conservation efforts and the well-being of all stakeholders in coastal communities.

## Supplementary Information

Below is the link to the electronic supplementary material.

Supplementary file1 (PDF 332 KB)
