# Supplementary material for: Coastal cultural ecosystem services and adolescents’ subjective well-being
Source: Ambio. 2024 Jun 13;53(11):1561–73. doi: 10.1007/s13280-024-02043-2 (PMC11436554; doi:10.1007/s13280-024-02043-2)
Supplement: Supplementary file 1 — Supplementary file1 (PDF 332 KB) [file 13280_2024_2043_MOESM1_ESM.pdf]

Coastal Cultural Ecosystem Services and Adolescents' Subjective Well-being

David Cabana[1,2]*, Stefania Pinna[1, 3], Simone Farina[1,4,5], Daniele Grech[1], Nicholas Barbieri[1], Ivan Guala[1]

[1]IMC - International Marine Centre, Loc. Sa Mardini Torregrande, 09170, Oristano, Italy

[2] Climate Service Center Germany (GERICS), Helmholtz-Zentrum Hereon, Fischertwiete 1, D-20095 Hamburg, Germany.

[3]GREEN LEAF - Groupe de Recherche en Education à l'Environnement et à la Nature, Laboratory of Affective Ecology, University of Valle d'Aosta.

[4]Department of Integrative Marine Ecology, Stazione Zoologica Anton Dohrn - National Institute of Marine Biology, Ecology and Biotechnology, Genoa Marine Centre, Villa del Principe, Piazza del Principe 4, 16126 Genoa, Italy

[5]National Research Council, Institute for the Study of Anthropic Impacts and Sustainability in the Marine Environment (CNR-IAS), Loc. Sa Mardini, 09170 Torre Grande, OR, Italy

*Corresponding Author: Dr David Cabana, david.cabana@hereon.de

Word count: 4800
Citations: 66

Acknowledgements: This article is based on research undertaken as part of Interreg V-A Italy-France Maritime 2014-2020 Cooperation Program. Project "Gestione Integrata delle Reti ecologiche attraverso i Parchi e le Aree Marine - GIREPAM" (Asse 2 - Lotto 3 - PI 6C-OS 1).

Conflict of Interest: All authors of this paper declare that they have no conflicts of interest.

Ambio

Supplementary Information

This supplementary information has not been peer reviewed.

Title**: Coastal Cultural Ecosystem Services and Adolescents' Subjective Well-being**

1. **Survey**

The Sardinian Coast

Q1. About you and your visits to the coast

Age: ☐ 0-18 ☐ 19-35 ☐ 36-55 ☐ 55+ **Gender:** ☐ Male ☐Female ☐ Other ☐ Prefer not to say

Where do you live? Council____________ Town_____________

How often do you visit the coast?

☐ Daily ☐ At least once a week ☐ At least once a month ☐ At least once a year ☐ This is my first time
☐ Other [Please describe] _____________

Q2. Why do you visit the coast? [Please tick all that apply]

☐ Bird Watching ☐ Golf ☐ Swimming ☐ Wildlife Watching
☐ Cycling ☐ Jogging ☐ Visiting a place or amenity ☐ Water Sports
☐ Dog walking ☐ Photography ☐ Walking ☐ **Other** [Please describe]: ___________

Q3. What values do you associate with the Sardinian coast? [Please tick all that apply]

☐ Contact with nature ☐ Leisure ☐ Sanctuary ☐ **Other** [Please describe]: _________________
☐ Educational ☐ Physical exercise ☐ Scenic

Q4. Please give a bit more detail about the values you have ticked above

Q5. What is special about the Sardinian coast?

Q6. What influences the health of the coastal and marine environment in the Sardinian coast? Why is this happening?

Q7. Over the past 10 years, have you noticed any changes in the coastal environment? If so, when and why did this happen?

Q8. Have any of these changes affected the way you use the Sardinian coast? *(about question 7)*

Table 1. Questions analysed for completing the different levels and components of the framework. The questions of the survey classify as: Open: fully open-end question. Close*: type of questions with pre-selected responses including the option to expand by adding elements and comments.

Question	Type	Contributing to feed the section of the framework
Q1. Age/Gender/ town of residence/ visiting frequency to the coast	Close*	General demographic information
Q2. Why do you visit the coast?	Close*	Cultural Practices
Q3. Which values do you associate with the Sardinian coastline?	Close*	Cultural Ecosystem Services (CICES)
Q4. Please give a bit more of detail about the values ticked before. *(about Q3)*	Open	Elements of the environment, HWB, cultural practices
Q5. What is special about the Sardinian coastline?	Open	Elements of the environment, HWB, cultural practices
Q6. What is influencing the health of the coastal and marine environment in Sardinia? Why is this happening?	Open	Drivers (Reshaping components), elements of the environment, HWB
Q7. Over the past 10 years, have you noticed any changes in the coastal environment? If so, when, and why did this happen?	Open	Drivers (Reshaping components), elements of the environment, HWB
Q8. Have any of these changes affected the way you use the Sardinian coastline? *(about Q7)*	Open	Elements of the environment, HWB
